# A Systematic Review of Clinical Practice Guidelines for Cataract: Evidence to Support the Development of the WHO Package of Eye Care Interventions

**DOI:** 10.3390/vision6020036

**Published:** 2022-06-20

**Authors:** Justine H. Zhang, Jacqueline Ramke, Chan Ning Lee, Iris Gordon, Sare Safi, Gareth Lingham, Jennifer R. Evans, Stuart Keel

**Affiliations:** 1 International Centre for Eye Health, London School of Hygiene and Tropical Medicine, London WC1E 7HT, UK; channing.lee@doctors.org.uk (C.N.L.); iris.gordon@lshtm.ac.uk (I.G.); jennifer.evans@lshtm.ac.uk (J.R.E.); 2 Royal Free Hospital, London NW3 2QG, UK; 3 School of Optometry and Vision Science, University of Auckland, Auckland 1142, New Zealand; 4 St Paul’s Eye Unit, Royal Liverpool University Hospital, Liverpool L7 8XP, UK; 5 Ophthalmic Research Center, Research Institute for Ophthalmology and Vision Science, Shahid Beheshti University of Medical Sciences, Tehran 16666-73111, Iran; saresafi.s@gmail.com; 6 WHO Collaborating Centre for the Eye Care and Prevention of Blindness, Tehran 16666-73111, Iran; 7 Centre for Ophthalmology and Visual Science, Lions Eye Institute, University of Western Australia, Perth 6009, Australia; garethlingham@lei.org.au; 8 Centre for Public Health, Queen’s University Belfast, Belfast BT7 1NN, UK; 9 Vision and Blindness Prevention Programme, World Health Organization, 1211 Geneva, Switzerland; keels@who.int

**Keywords:** cataract, clinical practice guidelines, eye care interventions

## Abstract

The World Health Organization (WHO) is developing a Package of Eye Care Interventions (PECI) to facilitate the integration of eye care into Universal Health Coverage. This paper presents the results of a systematic review of clinical practice guidelines for cataract in adults, to help inform PECI development. We searched academic and guideline databases, and websites of professional associations, for guidelines published between January 2010 and April 2020. Guidelines were excluded if there was commercial funding or unmanaged conflicts of interest. Quality appraisal was conducted using the *Appraisal of Guidelines for Research and Evaluation* (AGREE) II tool. We identified 3778 reports, 35 related to cataract guidelines, four of which met the inclusion criteria (United Kingdom: 2, United States: 1, Iran: 1). The recommendations across the four guidelines covered pre-operative (43%), intra-operative (37%), and post-operative interventions (20%). Most ‘strong’ recommendations were supported by good quality evidence. Differences in recommendations across guidelines may be attributable to time of publication or regional differences in surgical practice. Few guidelines met the quality criteria, and only three countries were represented. The results of this step of the PECI development process will inform subsequent phases for development of the WHO’s package of evidence-based eye care interventions for cataract.

## 1. Introduction

In its inaugural *World report on vision* in 2019, the World Health Organization (WHO) recommended that eye care should be integrated into Universal Health Coverage (UHC) to improve access to eye care services and strengthen eye care in health systems globally [1]. In response to this recommendation, the WHO is developing a priority package of evidence-based eye care interventions (PECI) in collaboration with Cochrane Eyes and Vision [2]. The development of PECI is in four phases: (1) selection of eye conditions; (2) identification of evidence-based eye care interventions using systematic methods to identify and appraise Clinical Practice Guidelines (CPGs); (3) agreement on interventions, their service delivery platforms, and the resources required for each intervention; (4) peer review. The PECI will be widely disseminated as a resource for countries to plan and integrate eye care interventions into eye care services across their population. 

It was estimated that in 2020, 94 million people globally had moderate or severe vision impairment (MSVI) or blindness due to cataract [3]. This treatable condition is the leading cause of blindness globally, and the second leading cause of MSVI [3]. In 2021, the World Health Assembly endorsed a resolution for all countries to increase their effective cataract surgical coverage this decade, which will require improvements in both access to services and the quality of those services [4,5,6]. 

Treatment of cataract generally only requires one operation to remove the cataract and place an artificial lens into the eye, along with relatively short pre- and post-operative assessments. Cataract surgery is safe and effective when delivered by competent teams in enabling environments. Phacoemulsification surgery is the most widely used procedure in high-income settings and has the best surgical outcome in terms of unaided vision; although, in terms of best corrected vision, the surgical outcomes between phacoemulsification and small incision cataract surgery (SICS) are similar [7,8]. SICS is faster and cheaper than phacoemulsification, and thus is currently more widely used in low-income settings [9]. 

The cataract surgical rate of a country (the number of cataract operations performed in one year, per million population) is correlated with economic indicators such as Gross Domestic Product (GDP), with increased surgical output increasing with GDP [10]. This suggests that delivery of cataract surgery is strongly dependent on resource availability. Therefore, achieving UHC is likely to be highly dependent on improving resource availability and access to high-quality cataract services in low- and middle-income settings. 

This paper aims to present the results, including quality appraisal, of a systematic review of CPGs for cataract in adults (Phase 2 of the PECI development process). 

## 2. Materials and Methods

We followed the Preferred Reporting Items for Systematic reviews and Meta-Analyses (PRISMA) reporting guidelines (Supplementary Materials S1) [11]. The rationale and methodology for the development of the PECI, including the systematic review of CPGs, is presented and published in a separate paper [2].

*Eligibility Criteria:* We included CPGs relating to cataract, from any country, published after 2010 in the English language. Full exclusion criteria for each stage of selection of the CPGs are provided in Table 1. 

*Information Sources and Search:* Systematic literature searching of MEDLINE, Embase, CINAHL, Global Health, Global Index Medicus was conducted by an experienced Cochrane Eyes and Vision information specialist (IG) in April 2020. The full search strategy can be found in Supplementary Materials S2. Where applicable, MeSH terms were used. We also conducted a search of relevant CPGs in guideline databases and the websites of professional ophthalmology and optometry associations (listed in Supplementary Materials S3). 

*Selection of Sources of Evidence and Appraisal:* Titles and abstracts were independently screened by two investigators (GL/SS), with the aid of a web-based organization tool (Abstrackr, available at: http://abstrackr.cebm.brown.edu, (accessed on 15 May 2022)). Any conflicts were resolved by discussion between a WHO (SK) and Cochrane Eyes and Vision (JE) representative. 

Full texts were then independently screened by two investigators (JZ/JR and JZ/CNL). Any conflicts were resolved by discussion; if the two investigators could not come to a consensus, the conflict was resolved by discussion with a third investigator (SK). 

Quality appraisal of CPGs was conducted independently by two investigators (JZ/JR) using the *Appraisal of Guidelines for Research and Evaluation II* (AGREE II) tool [12]. Specific items in the AGREE II tool were used for the selection of CPGs, being those agreed by researchers to be the most relevant to selecting high-quality CPGs in a previous WHO package of interventions [13], and in accordance with the protocol [2] (items 4, 7, 8, 10, 12, 13, 15, 22 and 23; Table 2). Each item was scored on a 7-point Likert scale, from 1–7 (*strongly disagree* to *strongly agree*), so the maximum possible total score for all 9 items was 63. If the scores given by the two investigators differed by more than 2, the conflict was resolved by discussion between the two investigators, or with a third investigator. CPGs were excluded if: (1) the average score between the two investigators was less than 3 for items 4, 7, 12, or 22; or (2) the sum of the average score of the two investigators was less than 45 for items 4, 7, 8, 10, 12, 13, 15, 22, and 23. 

*Data Charting and Data Items:* Data were charted using a standardized form. Data from each CPG were extracted by a single investigator (JZ) and verified by a second investigator (JR). Any conflicts were resolved by discussion with a third investigator (SK). The following data items were extracted:

1. Title of the CPG;
2. Sponsoring organization (if there was no organization, then the name of the first author was extracted);
3. Publication year;
4. Title of the chapter to which the recommendation refers to;
5. Page number, title and numbering of the section in which the recommendation is stated;
6. Intervention target;
7. Intervention category;
8. Intervention name;
9. Who usually provides the intervention;
10. Dosage or frequency of the intervention;
11. The specific recommendation copied and pasted from the relevant paragraph in the CPG;
12. Recommendation strength, and the name or description of the classification system used;
13. Quality or level of evidence relating to the recommendation, and the name or description of the classification system used;
14. Any other remarks on the recommendation that the investigator believes to be relevant.

*Synthesis of Results:* Recommendations of eye care interventions were tabulated, and categorized into pre-operative, intra-operative, and post-operative recommendations. For each intervention tabulated, the corresponding CPG(s) that recommended that intervention was listed, alongside judgements on the quality of evidence and strength of recommendation. 

As the CPGs used different frameworks for assessing the quality of evidence and strength of recommendation, we present the results narratively for each CPG. 

*Ethics and Dissemination:* As this study only included freely available CPGs, ethics approval was not sought. Results will be widely disseminated after the WHO, supported by a Technical Advisory Group of experts, have agreed on which interventions should be included in the final PECI (Phase 3), and after the peer review process (Phase 4).

## 3. Results

**Figure 1:**
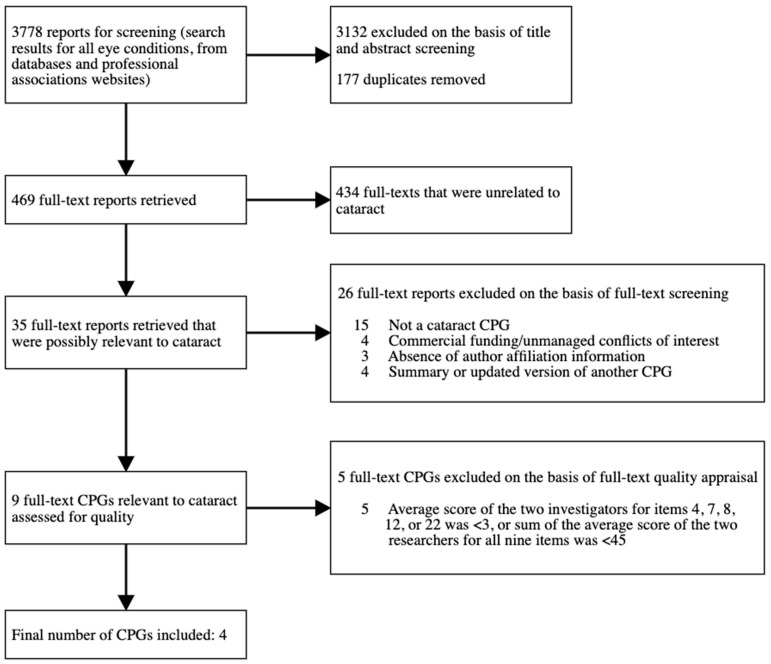
Flow chart of the screening process.

After combining all searches (academic and guideline databases, and website searches of professional associations), 3778 reports were identified. Then, 177 duplicates were removed, and 3132 were excluded after title and abstract screening. Finally, 469 full text reports were retrieved, of which 35 were related to cataract. Of the 35 reports, 26 were excluded after full text screening, and nine CPGs went through to the appraisal stage, five of which were excluded (Figure 1; Table 3). This left four CPGs eligible for inclusion. The median AGREE II score for the four selected CPGs was 49.4 (range 45–53.5), while the median score of the five excluded CPGs was 17.2 (range 12–29.5). The results of the screening process and exclusion reasons are summarized in Figure 1. 

The following four CPGs were included:

1. National Institute for Health and Care Excellence (NICE), 2017, Cataracts in adults; management [14].
2. American Academy of Ophthalmology, 2016, Cataract in the Adult Eye Preferred Practice Pattern [15].
3. Rajavi et al., 2015, Customized clinical practice guidelines for management of adult cataract in Iran [16].
4. The Royal College of Ophthalmologists and Clinical Council for Eye Health Commissioning, 2018, Commissioning guide: Adult cataract surgery [17].

CPGs 1 and 4 were published by organizations based in the United Kingdom, CPG 2 is from the United States, and CPG 3 is from Iran. CPGs 1 and 2 drew evidence directly from original research (such as systematic reviews, RCTs, and observational studies), whereas CPG 3 drew evidence from other CPGs (namely the American Academy of Ophthalmology’s 2006 and 2011 CPGs, Canadian Ophthalmological Society’s 2008 CPG, and the Royal College of Ophthalmologists’ 2010 CPG). CPG 4 drew evidence from a mixture of original research and other organizations’ CPGs (such as NICE guidelines). 

Only CPGs 1–3 reported the quality of evidence supporting their recommendations (Table 4). CPGs 1 and 2 used GRADE to report quality ratings [18]: In CPG 1, 28% of recommendations were supported by high-quality evidence, whilst 50% of recommendations were supported by low-quality evidence; in contrast, CPG 2 judged that 86% of recommendations were supported by good quality evidence and only 3% of the recommendations were supported by insufficient evidence. CPG 3 used its own method of reporting quality (Table 4 legend) and found that 38% of the recommendations were supported by evidence at the level of a randomized clinical trial, systematic review or meta-analysis. 

Only CPGs 1 and 2 reported the strength of recommendation (Table 4). For CPG 1, three quarters of the recommendations were *strong*, and the other one quarter of their recommendations were *discretionary* (where the trade-offs between benefits and risks were less certain). For CPG 2, 97% of the recommendations were *strong*, with only 3% reported as *discretionary*. 

A summary of the eye care intervention recommendations from the four CPGs are presented in Appendix A Table A1. After grouping the recommendations, 65 recommendations related to pre-operative interventions, 55 recommendations related to intra-operative interventions, and 30 related to post-operative interventions. 

Most of the strongly recommended interventions had good quality evidence supporting them, for example, weighing up risks and benefits of surgery with the patient, use of appropriate biometry, small incision surgery, prepping with povidone-iodine, and appropriate counselling of the patient regarding complications and post-operative care. 

A minority of interventions were strongly recommended without high quality evidence. For example, no evidence was identified for the recommendation to offer eye protection for patients whose eye shows residual effects of anesthesia at time of discharge after surgery.

There are instances where the CPGs make recommendations that do not align. For example, CPGs 1 and 4 indicate that immediate sequential bilateral cataract surgery (ISBCS) (i.e., bilateral cataract surgery performed in the same session) can be considered in low-risk patients; whereas CPG 3 recommends that surgery should be performed in separate sessions in patients with bilateral cataract.

## 4. Discussion

Our systematic review found four CPGs relating to cataract that met our inclusion criteria. In the three CPGs where quality of evidence was evaluated, high-quality evidence was available for 18% (CPG 2), 86% (CPG 1) and 38% (CPG 3) of the interventions recommended. In the two CPGs where strength of recommendation was presented (CPGs 1 and 2), 75% and 97% of interventions were strong recommendations, respectively. The recommendations were broad in scope, covering pre-operative (43%), intra-operative (37%), and post-operative interventions (20%). 

A minority of interventions were strongly recommended without high quality evidence. However, in some cases, conducting a study might be unethical or impractical. For example, it would be unethical to randomize patients to a study arm where they are not offered eye protection, as this could lead to complications such as corneal abrasions. Since eye protection (e.g., plastic eye shields) is inexpensive, has minimal risk of causing harm and could avert complications, it seems reasonable to make a strong recommendation for eye protection despite the lack of evidence. Additionally, it would be unethical to perform a trial comparing cataract surgery versus no cataract surgery (as this would leave some participants visually impaired). Therefore, while there are research and trial data comparing different cataract procedures, there is no evidence from randomized controlled trials for cataract surgery per se. 

The four included CPGs were published between 2015 and 2018. However, CPG 3 itself sourced its recommendations from four other CPGs that were published between 2006 and 2011 [16]: the American Academy of Ophthalmology’s 2006 and 2011 CPGs [15,19], the Canadian Ophthalmological Society’s 2008 CPG [20], and the Royal College of Ophthalmologists’ 2010 CPG [21]. The Royal College of Ophthalmologists’ 2010 CPG is now ‘archived’ and no longer in use. The American Academy of Ophthalmology’s 2006 and 2011 CPGs have now been replaced by the updated 2016 version, which is included in this systematic review (CPG 2). Thus, the recommendations outlined in CPG 3 are based on older CPGs, so CPG 3 may contain recommendations that are no longer in current best practice. For example, CPG 3 indicates that intracameral antibiotics are not recommended, which is not in line with recommendations from CPGs 1 and 2 that support the use of an intracameral antibiotic, cefuroxime. 

There was a consistent view across the CPGs that, pre-operatively, patients should be given adequate information to help them weigh up the risks and benefits of cataract surgery, and the patient’s individual preferences and needs should be considered. Use of biometry and keratometry prior to cataract surgery was emphasized by three of the four CPGs. Intraoperatively, there was a consistent view that the preferable method for cataract surgery is small incision surgery under local anesthesia, and the patient’s eyes should be prepped with povidone-iodine. Post-operatively, it was recommended that patients are given adequate information regarding their post-operative care, and topical steroids or non-steroidal anti-inflammatory drugs should be offered to reduce post-operative inflammation, particularly in patients at risk of cystoid macular oedema. Two CPGs also recommended that providers of cataract services should be able to provide commissioners with data to monitor quality, outcomes, and adverse events. Finally, counselling patients regarding the risk factors for developing cataracts, such as smoking, sun exposure, and long-term steroid use, was recommended by two CPGs. 

There are other instances where the CPGs make recommendations that are somewhat conflicting. For example, there are conflicting recommendations regarding ISBCS. Whilst there is evidence supporting ISBCS [22,23], surgeon willingness to perform ISBCS varies, which may be due to regional differences in surgical practice. For example, ophthalmologists in the UK cite the lack of Royal College and medico-legal approval for ISBCS as a barrier to performing ISBCS [24]. In addition, attitude to, and acceptance of, ISBCS has changed over time [25], and earlier CPGs may have been more reluctant to recommend ISBCS compared to more recent CPGs due to lower acceptance of ISBCS at the time. Ongoing systematic reviews and accumulation of evidence over time are likely to resolve this uncertainty [26].

Very few CPGs met the inclusion criteria, which is a surprising finding given that cataract is such a common condition, and in many settings, cataract surgery is one of the most frequently performed operations [27]. Four CPGs were excluded due to unmanaged conflicts of interest. We recommend that in future, organizations preparing CPGs develop a clear and transparent approach to the management of conflicts of interest. We would also recommend that future CPGs draw on the *Appraisal of Guidelines for Research and Evaluation II* (AGREE II) tool [12], to ensure the quality and structure of their CPG before distributing it for wider use. 

A limitation of our methodology is that we restricted our search to English language papers only, and thus may have missed CPGs that are published in non-English speaking settings. This decision was due to feasibility considerations, including the labour-intensive nature of screening and translation of CPGs in all official WHO languages. Another limitation is that some CPGs were excluded based on an absence of information regarding author affiliation, as lack of author affiliation does not necessarily mean that a conflict of interest is present. Additionally, the publication year of the CPG may not reliably reflect how up-to-date the cited evidence was. For instance, CPG 3 drew on evidence from other published CPGs, half of which were published prior to 2010. Given that cataract surgery is a long-standing, well-established and cost-effective intervention, this may limit the amount of new evidence in this field. As only four CPGs were identified, and only one was from a low/middle-income country (LMIC), the recommendations drawn from these four CPGs may only be applicable in certain settings. 

Due to timeline constraints for the development of the PECI, the literature search was conducted in April 2020, so CPGs published subsequently were not included in this study. We may have missed more recent guidelines that can be considered in future updates to the PECI, such as “Cataract—Treatment of Adults, Ministry of Health British Columbia, 2021” [28], and “Cataract in the Adult Eye Preferred Practice Pattern, American Academy of Ophthalmology, 2021” [29]. Additionally, the global COVID-19 pandemic, which was declared by the WHO in 2020 [30], had a major impact on clinical practice. For example, some updated guidelines, such as “Management of Cataract in India Revised in August 2020, VISION 2020: The Right to Sight—INDIA” [31], recommended organizational changes in operating theatres. These guidelines should be considered in future PECI updates. 

Among the cataract CPGs from LMIC settings, only one CPG from Iran met the inclusion criteria for our review. Cataract recommendations in LMIC settings may differ from those in high-income settings; for example, resource constraints in some countries may mean that recommendations cover how to prioritize patients for cataract surgery according to level of need. To address this, a broad range of public health, academic and clinical professionals from all WHO regions, with a particular focus on LMIC settings, were involved in all stages of development of the PECI. There is a pressing need for more high-quality CPGs from LMIC settings, particularly as cataract is highly prevalent in these regions.

## 5. Conclusions

The results presented in this paper will assist in informing Phases 3 and 4 of the PECI development process, with the ultimate goal of developing a package of evidence-based eye care interventions for cataract [2]. 

## Figures and Tables

**Table 1:** Exclusion criteria for screening of Clinical Practice Guidelines (CPGs).

Title and Abstract Screening	Full Text Screening	Quality Appraisal *
(1) The identified report was not a CPG (2) The guideline was published before 2010 (3) The guideline was not in English (4) The guideline was not developed for cataract	(1) There was commercial funding or unmanaged conflicts of interest (2) Absence of affiliation of authors	(1) The average score of the two investigators for items 4, 7, 8, 12, or 22 was below 3. (2) the sum of the average score of the two investigators was less than 45 for items 4, 7, 8, 10, 12, 13, 15, 22, and 23.

* Items and scores refer to the *Appraisal of Guidelines for Research and Evaluation II* (AGREE II) tool [12].

**Table 2:** AGREE II tool items used for the selection of Clinical Practice Guidelines (CPGs).

AGREE II Item Number	Item Description
4	The guideline development group includes individuals from all relevant professional groups *
7	Systematic methods were used to search for evidence.
8	The criteria for selecting the evidence are clearly described.
10	The methods for formulating the recommendations are clearly described.
12	There is an explicit link between the recommendations and the supporting evidence.
13	The guideline has been externally reviewed by experts prior to its publication.
15	The recommendations are specific and unambiguous.
22	The views of the funding body have not influenced the content of the guideline.
23	Competing interests of guideline development group members have been recorded and addressed.

* For this manuscript, relevant professional groups include ophthalmologists, optometrists, orthoptists, eye health researchers, and other allied eye health professionals.

**Table 3:** AGREE II scores for the Clinical Practice Guidelines (CPGs) that were selected for appraisal after full text screening.

Clinical Practice Guideline (CPG)	Average AGREE II Scores					
	Item Number(s)					
	4	7	8	12	22	4, 7, 8, 10, 12, 13, 15, 22, 23
*CPGs selected for inclusion after appraisal*						
National Institute for Health and Care Excellence (NICE), 2017, Cataracts in adults; management [14]	7	7	7	7	7	53.5
American Academy of Ophthalmology, 2016, Cataract in the Adult Eye Preferred Practice Pattern [15]	5	6.5	4	6	7	49.5
Rajavi et al., 2015, Customized clinical practice guidelines for management of adult cataract in Iran [16]	5	3	4	6.5	6.5	49.5
The Royal College of Ophthalmologists and Clinical Council for Eye Health Commissioning, 2018, Commissioning guide: Adult cataract surgery [17]	7	7	6	5	4.5	45
*CPGs excluded after appraisal*						
The Royal College of Ophthalmologists and the Royal College of Anaesthetists, 2012, Local Anaesthesia for Ophthalmic Surgery	6.5	3	5	3.5	1	29.5
The Royal College of Ophthalmologists, 2018, Ophthalmic Services Guidance: Theatre Procedures	2	1	1	1	1	15.5
The Royal College of Ophthalmologists, 2016, Ophthalmic Services Guidance: Managing an outbreak of postoperative endophthalmitis	2.5	1	1	1	1	14.5
The Royal College of Ophthalmologists, 2018, Ophthalmic Services Guidance: Theatre facilities and equipment	2	1	1	1	1	14.5
The Royal College of Ophthalmologists and the UK Ophthalmology Alliance, 2018, Quality Standard: Correct IOL implantation in cataract surgery	1	1	1	1	1	12

**Table 4:** Quality of evidence and strength of recommendation of the included Clinical Practice Guidelines (CPGs).

Clinical Practice Guideline	Quality of Evidence, n (%) *			Strength of Recommendation, n (%)	
	High/Good	Moderate	Low/Insufficient	Strong	Discretionary
CPG 1: National Institute for Health and Care Excellence (NICE), 2017, Cataracts in adults; management [14] **	6 (27)	5 (23)	11 (50)	36 (75)	12 (25)
CPG 2: American Academy of Ophthalmology, 2016, Cataract in the Adult Eye Preferred Practice Pattern [15].	64 (86)	8 (11)	2 (3)	72 (97)	2 (3)
CPG 3: Rajavi et al., 2015, Customized clinical practice guidelines for management of adult cataract in Iran [16] ***	I: 31 (38)	II: 11 (14)	III–IV: 39 (48)	-	-
CPG 4: The Royal College of Ophthalmologists and Clinical Council for Eye Health Commissioning, 2018, Commissioning guide: Adult cataract surgery [17] ***	-	-	-	-	-

* Definitions of quality: CPGs 1 and 2: high/good, moderate and low/insufficient quality ratings defined by GRADE [18]. CPG 3: I Randomized clinical trials; Systematic reviews; Meta-analysis. II Controlled clinical study without randomization at least one; Well-designed cohort study; Well-designed case–control; Cross sectional study. III Surveys; descriptive; case series studies. IV Experts opinion; consensus. ** Quality of evidence is based on 22 groups of recommendations, and strength of recommendation is based on 48 recommendations (some groups contain multiple recommendations). *** Strength of recommendations or quality of evidence not reported in the CPG.

## Data Availability

Data generated from this review will be available upon reasonable request from Justine Zhang (justine.zhang@lshtm.ac.uk).

## Supplementary Materials

The following are available online at https://www.mdpi.com/article/10.3390/vision6020036/s1, Supplementary Material S1: PRISMA reporting guidelines; Supplementary Material S2: Search strategy for academic databases; Supplementary Material S3: Guideline databases and the websites of professional ophthalmology and optometry associations searched.

## Appendix A

**Table A1:** Summary of the recommended interventions for cataract, identified from Clinical Practice Guidelines (CPGs) that met the inclusion criteria. The corresponding CPGs, highest level of quality of evidence, and the strength of recommendation are presented.

Description of Intervention	Relevant CPG(s) *	Quality of Evidence **	Strength of Recommendation (Strong, Discretionary) ***
***Pre-operative interventions***			
Cataract surgery is indicated in patients with cataracts causing vision loss, phacomorphic glaucoma, lens-induced uveitis, or posterior segment diseases where the cataract is limiting the retinal examination/treatment.	3	IV	-
Patients should be given oral and written information about cataract surgery, in an accessible format.	1	Moderate	Strong
	4	-	-
Weigh up the indications, risks, and benefits of cataract surgery with the patient.	1	High	Strong
	3	I	-
In patients with cataract in only one eye, surgery is recommended if the advantages outweigh the risks.	3	III	-
Do not restrict access to cataract surgery based on visual acuity; it should be based on individual need.	1	High	Strong
	4	-	-
For patients who require a certain visual acuity for their occupation, cataract surgery is indicated even when the patient does not experience functional deficits.	3	IV	-
Offer second eye cataract surgery using the same criteria as for first eye surgery.	1	Moderate to high	Strong
In some cases of anisometropia, earlier surgical intervention for the second eye is recommended, but the time between surgeries should be long enough to first eye surgical complications.	3	IV	-
In patients with bilateral cataract, it is recommended that surgery is performed in separate sessions to achieve better binocular vision.	3	I	-
In patients with bilateral cataract, it is recommended that surgery is performed in separate sessions due to the risk of endophthalmitis and toxic anterior segment syndrome.	3	III	-
Immediate sequential bilateral cataract surgery should be considered for people who are at low risk of operative and post-operative complications, and for people who require general anaesthesia. Potential benefits and risks should be fully discussed with patients pre-operatively.	1	Low to moderate	Discretionary
	4	-	-
Cataract surgery may reduce intraocular pressure in patients with angle closure glaucoma	3	II	-
Simultaneous cataract and glaucoma surgery is recommended if there is a risk of blindness due to increased intraocular pressure post-operatively.	3	IV	-
Use biometry (preferably optical biometry over ultrasound biometry) and keratometry to estimate axial length and central corneal curvature, respectively.	1	Low	Strong
	2	III, good quality	Strong
	3	I	-
Repeat A-scan biometry if: the axial length is >26 mm or <21 mm; keratometry is >47 D or <41 D; astigmatism is >2.5 D; axial length difference between the two eyes is >0.7; keratometry difference between the two eyes is >0.9.	3	III	-
Consider corneal topography for people with irregular astigmatism or previous refractive surgery	1	Low	Discretionary
For patients with previous refractive surgery, use adjusted formulas to calculate the intraocular lens power. Advise them that refractive outcomes after cataract surgery are difficult to predict.	1	Very low to moderate	Strong
	3	IV	-
Documentation should be kept by the treatment centre for all patients undergoing refractive surgery.	3	II	-
Surgeons should consider optimizing a manufacturer’s recommended intraocular lens constant, e.g., based on their previous refractive outcomes.	1	low	Strong
	3	IV	-
Consider using the first-eye refractive outcome to guide calculations for the intraocular lens power for second-eye cataract surgery.	1	Very low to low	Discretionary
In eyes with abnormal size, use Holladay 2 or Haigis formulas.	3	IV	-
Use new generation formulas to calculate the intraocular lens power.	3	III	-
Patients should be informed of the potential inaccuracy of intraocular lens power calculations and that further surgery may be required to achieve the target refractive outcome.	2	III, good quality	Strong
Consider using a validated risk stratification algorithm to identify people at increased risk of complications during and after surgery.	1	Low	Discretionary
Explain the results of risk stratification to the patient and discuss how it may affect decision-making.	1	Low	Strong
In cases where the cataract surgery is likely to be complex, and there may be a high risk of complications, or the surgeon is not experienced enough, the surgery should be performed by a more experienced surgeon or the patient should be referred to a facility with more expertise.	3	II	-
If a retinal detachment is found on pre-assessment, consider combined vitrectomy and cataract surgery.	3	III	-
In patients with Fuchs’ dystrophy and cataract, combined cataract and corneal transplant can be considered.	3	IV	-
If possible, phacoemulsification should be performed before penetrating keratoplasty if visualization is adequate.	2	III, good quality	Strong
Explain to people who are at risk of developing dense cataracts that delayed surgery can result in an increased risk of complications.	1	Low	Strong
	2	III, good quality	Strong
Do not offer multifocal intraocular lenses for patients with cataracts.	1	Very low to moderate	Strong
The suitability of multifocal intraocular lenses for patients with amblyopia, or abnormalities of the cornea, optic nerve or macula must be carefully considered.	2	III, insufficient quality	Discretionary
Offer monovision to patients with anisometropia, or pre-operative monovision.	1	Very low to moderate	Strong
Assess tear function, as tear dysfunction may compromise the postoperative result.	2	II+, good quality	Strong
Counsel patients to stop smoking.	2	II+, good quality	Strong
	3	I	-
Recommend brimmed hats and ultraviolet-B blocking sunglasses.	2	II-, good quality	Strong
	3	I	-
Recommend safety eyeglasses in high-risk activities at work or recreation.	2	III, good quality	Strong
Advise patients that currently there is insufficient evidence to support the use of pharmacological treatments for cataract.	2	III, good quality	Strong
Advise patients that long-term use of steroids is associated with increased risk of cataract.	2	II+, moderate quality	Strong
	3	I	-
Vitamin supplements do not reduce the progression of cataracts.	3	I	-
Increased risk of cataracts should be discussed with diabetic patients.	3	II	-
In patients with cataracts who do not want surgery, the increased risk of accidents and bone fractures should be discussed.	3	II	-
Patients with cataracts should undergo surgery as soon as possible, preferably with a waiting time of less than 2–3 months to avoid possible falls, fractures and accidents.	3	I	-
In a functionally monocular patient, tell the patient that blindness is one of the risks of cataract surgery.	2	III, good quality	Strong
Patients should be informed pre-operatively regarding the possibility of visual impairment continuing after surgery.	2	III, good quality	Strong
A medical pre-operative evaluation should be performed by the surgeon, and where appropriate, the primary care physician, to determine the appropriateness and timing of surgery.	2	III, good quality	Strong
	3	I	-
Pre-operative laboratory testing is not indicated.	2	I+, good quality	Strong
Consider the patient’s preferences and needs when selecting the post-operative refractive target.	1	Moderate	Strong
	2	III, good quality	Strong
α-1 antagonists should be discontinued before surgery due to the high risk of floppy iris syndrome.	3	II	-
Anti-coagulant and anti-platelet medications should generally be continued.	2	I-, good quality	Strong
For patients on warfarin, the international normalized ratio should be in the therapeutic range.	2	I+, good quality	Strong
Aspirin should only be discontinued peri-operatively if the risk of bleeding outweighs its potential benefit.	2	I-, good quality	Strong
Discontinuation of anti-coagulant medications is not recommended, except for warfarin and clopidogrel.	3	I	-
Documentation of which eye is being operated on, IOL power, medications, previous diseases, etc, should be filled in immediately before surgery.	3	II	-
All patients with cataracts should be informed about the risk of posterior capsular opacification before undergoing surgery.	3	III	-
The risk of aggravation of diabetic retinopathy should be discussed in patients with diabetes who are undergoing cataract surgery.	3	III	-
If possible, treatment of proliferative diabetic retinopathy and macular oedema should be carried out before cataract surgery.	3	IV	-
Risk of retinal detachment should be discussed with high-risk patients, e.g., young, male, high myope.	3	I	-
Patients with age-related macular degeneration should only undergo cataract surgery is there is a chance for improved vision, and the patient should be informed of the risk of worsening of their macular degeneration.	3	I	-
Patients should be counselled prior to surgery if they have a comorbidity that is associated with intra-ocular complications or the potential for reduced improvement in visual function.	2	III, good quality	Strong
In patients with uveitis, inflammation should be at its best level of control, for ≥3 months prior to elective surgery, and anti-inflammatory medications should be started prior to surgery. The medical regimen should be individualized, based on the severity of previous episodes of uveitis.	2	III, good quality	Strong
In patients with uveitis, surgical planning should take into account the possible need for further procedures secondary to uveitis complications, e.g., secondary glaucoma.	2	III, good quality	Strong
Ensure that the correct medical notes are used by confirming the patient’s name, address and date of birth, and ensure that biometry results are securely attached to the patient’s notes. Record the patient’s choice of refractive outcome in the medical notes.	1	Moderate	Strong
Staff in the cataract pathway should be able to provide evidence of competencies and continuing professional development.	4	-	-
While certain diagnostic procedures may be delegated to appropriately trained staff supervised by the ophthalmologist, interpretation of these procedures requires the clinical judgement of the ophthalmologist.	2	III, good quality	Strong
***Intra-operative interventions***			
The predominant method of cataract surgery in high income countries is small incision phacoemulsification with foldable intraocular lens implantation.	2	I+, good quality	Strong
	3	I	-
Small incision surgery is generally preferred.	2	I-, good quality	Strong
Only an ophthalmologist has the medical and microsurgical training as part of a comprehensive resident experience needed to perform cataract surgery.	2	III, good quality	Strong
Ensure that surgeons in training are well supervised.	1	Low	Strong
Local anaesthesia is preferred, but in some cases general anaesthesia is indicated.	2	I++, good quality	Strong
Offer sub-Tenon’s or topical anaesthesia.	1	Very low to high	Strong
Consider hyaluronidase as an adjunct to sub-Tenon’s anaesthesia	1	Low	Discretionary
If both sub-Tenon’s and topical are contraindicated, consider peribulbar anaesthesia.	1	Very low to high	Discretionary
Do no offer retrobulbar anaesthesia.	1	Very low to high	Strong
Consider sedation as an adjunct to anaesthesia for people who are anxious, have postural problems, or where surgery is expected to take longer than usual.	1	Low	Discretionary
There is insufficient evidence to recommend sedation over local anaesthesia.	2	I+, good quality	Strong
When sedation is used, intravenous access is recommended.	2	I+, good quality	Strong
The choice of local anaesthesia is based on patient and surgeon preference.	2	I+, good quality	Strong
	3	I	-
Monitoring during administration of anaesthesia generally includes a heart monitor, pulse oximetry, blood pressure and respiratory rate, performed by a qualified staff member.	2	III, good quality	Strong
Before giving anaesthesia, use a WHO surgical safety checklist to check the patient’s identity, consent form, printed biometry results, medical notes, preferred refractive outcome, lens, marked eye to be operated on, and consistency of lens formulas and calculations.	1	Moderate	Strong
Ensure there is only one matching intraocular lens in the theatre, and that alternative intraocular lenses (e.g., noncapsular-bag lenses) are in stock in the event of surgical complications.	1	Moderate	Strong
	2	III, good quality	Strong
As non-capsular bag fixation may increase the potential for optic tilt/decentration, the surgeon should consider whether multifocal intraocular lenses or lenses with higher degrees of negative spherical aberration should be used.	2	III, insufficient quality	Discretionary
A peripheral iridectomy should be performed to prevent the risk of pupillary block associated with an anterior chamber intraocular lens.	2	III, good quality	Strong
Consider on-axis surgery or limbal-relaxing incisions to reduce astigmatism.	1	Moderate	Discretionary
Only use femtosecond laser-assisted cataract surgery as part of a randomized control trial.	1	Low	Strong
There are certain types of cataracts, e.g., posterior polar, for which the femtosecond laser should not be used.	2	II-, moderate quality	Strong
In patients at risk of floppy iris syndrome, consider intracameral phenylephrine to increase pupil size.	1	Low	Discretionary
Follow a protocol for posterior capsule rupture that covers vitreous removal from the anterior chamber, minimizing retinal traction, lens fragment and soft lens matter removal, and implications of intraocular lens insertion.	1	No evidence was identified	Strong
Do not use capsular tension rings in routine cataract surgery.	1	High	Strong
Consider use of capsular tension rings in patients with pseudoexfoliation.	1	High	Discretionary
Use pre-operative anti-septics, and commercially or pharmacy prepared intracameral cefuroxime, to prevent endophthalmitis.	1	Very low to high	Strong
	2	I-, good quality	Strong
Intracameral antibiotic injections are not recommended due to the toxic effects and likelihood of reduction in corneal endothelial cells.	3	III	-
Discourage use of antibiotic in the irrigation bottle.	2	III, moderate quality	Strong
Construct and close incisions so that they are watertight, to reduce risk of infection.	2	II-, moderate quality	Strong
Eyelashes should be prepped with 10% povidone-iodine.	3	II	-
The conjunctiva should be prepped with 5% povidone-iodine.	2	II-, moderate quality	Strong
	3	II	-
In immediate sequential bilateral cataract surgery, the second eye should be treated as the eye of a different patient would be treated, using separate prepping, draping, instrumentation, irrigation solutions, and medications.	2	III, good quality	Strong
In patients with planned immediate sequential bilateral cataract surgery, if a complication occurs during first eye surgery, then second eye surgery should be reconsidered and carried out at a later date.	2	III, good quality	Strong
The surgeon should avoid working close to the cornea.	2	III, good quality	Strong
The surgeon should ensure proper orientation of the intraocular lens.	2	III, good quality	Strong
If there is vitreous loss, the surgeon should perform an anterior vitrectomy and implant an intraocular lens with appropriate size and design.	2	III, good quality	Strong
In patients with uveitis, excessive iris manipulation should be minimized and post-operative short-acting topical mydriatic agents may help prevent synechiae formation. Adjunctive steroids at the time of surgery should be considered.	2	III, good quality	Strong
Use intraocular lenses with sharp angles to reduce the risk of posterior capsule opacity.	3	I	-
Use of aspheric intraocular lenses is recommended to achieve better contrast sensitivity and visual performance; but in conditions such as zonular rupture, astigmatism, or after hyperopic corneal refractive surgery, spherical intraocular lenses should be implanted.	3	I	-
In the process of using multifocal intraocular lenses, careful patient selection, consultation, and utilization of preoperative examinations are vital.	3	IV	-
Base the use of multifocal/adjustable lenses on patient needs and desires, and after giving the patient information about the advantages and disadvantages.	3	I	-
To use toric intraocular lenses in patients with astigmatism, accurate preoperative calculations, correct marking of the steep axis, and implanting the intraocular lenses in correct axis should be considered.	3	IV	-
Visual outcomes after implantation of UV filter intraocular lenses and blue filter intraocular lenses are comparable.	3	I	-
When the posterior chamber intraocular lenses is placed in the ciliary sulcus, decreasing the power by 0 to 1.5 dioptres should be considered.	3	IV	-
Staining of the anterior capsule is recommended in mature cataracts, complicated cataracts and paediatric cataracts.	3	I	-
A smaller capsulorhexis (4.5–5 mm) is preferable to a larger one since it decreases the chance of posterior capsule opacification.	3	I	-
It is recommended that hydrodissection and hydrodelineation be performed to reduce tension on the zonules, facilitate cortex removal, and reduce the chance of posterior capsule opacification.	3	I	-
Floppy iris syndrome can be anticipated in patients using oral α-1 antagonists, and pharmacologic approaches, viscomydriasis, and pupil-expansion devices should be used to manage floppy iris syndrome.	2	II-, moderate quality	Strong
	3	I	-
A small pupil should be enlarged, e.g., with hooks/rings.	3	IV	-
Performing phacoemulsification with a torsional probe is preferable to longitudinal probes since there is a smaller risk of corneal trauma.	3	I	-
In patients with zonular weakness, applying capsular tension rings is recommended.	3	I	-
It is recommended to implant intraocular lenses using injectors.	3	III	-
In the absence of inadequate capsular bag support, the surgeon should determine a suitable intraocular lens to be implanted into the ciliary sulcus. Implantation of multifocal and aspheric intraocular lenses in the ciliary sulcus are not recommended.	3	III	-
In cases of posterior capsular rupture and inadequate capsular support for in-the-bag intraocular lens implantation, anterior chamber intraocular lenses, scleral fixation posterior chamber intraocular lenses, or iris fixation intraocular lenses may be used.	3	III	-
Subconjunctival antibiotic injection may be recommended to reduce the chance of postoperative endophthalmitis.	3	III	-
***Post-operative interventions***			
The operating ophthalmologist should provide aspects of post-operative care that are within their competence, and should inform patients about the symptoms of possible complications and details regarding their post-operative care. This includes instructions to notify the ophthalmologist promptly if problems occur.	2	III, good quality	Strong
At the first review appointment after cataract surgery, give patients information about eye drops, what to do if their vision changes, who to contact for queries, when to buy new spectacles, second eye cataract surgery, how to manage ocular comorbidities.	1	Low	Strong
It is recommended to perform the first post-operative examination up to 24 h after the surgery. The exact timing of other postoperative visits depends on surgical complications, and surgeon and patient preference.	3	IV	-
In high risk conditions such as monocular patients, glaucomatous eyes and surgical complications, the first post-operative visit should be performed within 24 h and more frequent follow-up examinations are needed.	3	IV	-
Patients should always have access to an ophthalmologist.	2	III, good quality	Strong
A final refractive visit should be made to provide a prescription for spectacles.	2	III, good quality	Strong
Final evaluation of refractive power should be performed 2 weeks post-operatively in patients with small corneal incisions (under 3.5 mm) and 6 weeks post-operatively in patients with larger incisions or extracapsular surgery.	3	IV	-
If a surgeon encounters a higher incidence of endophthalmitis compared to what is reported in the literature, the source of this should be investigated by taking microbial cultures from the personnel, surgery room and devices.	3	IV	-
Patient education brochures should be given to patients after surgery.	3	II	-
Providers of cataract care should be able to provide commissioners with outcome data, and outcome data from primary care should be fed back to the surgical team. Providers and surgeons should use audit tools to monitor quality, outcomes and adverse events in real time.	1	Low	Strong
	4	-	-
The surgical facility should comply with local and state regulations and standards.	2	III, good quality	Strong
Costlier new infection control measures that do not have evidence-based support should not be arbitrarily imposed by regulatory agencies.	2	III, good quality	Strong
If a wrong lens is implanted, refer to NHS England’s Never Events policy, undertake a root-cause analysis, and establish strategies and implementation tools to prevent future occurrences.	1	Moderate	Strong
Offer topical steroids or non-steroidal anti-inflammatory drugs after cataract surgery in patients at risk of cystoid macular oedema.	1	Very low to low	Strong
	3	I	-
Consider topical steroids in combination with non-steroidal anti-inflammatory drugs after cataract surgery in patients at risk of cystoid macular oedema.	1	Very low to low	Discretionary
Intraocular pressure should be monitored in patients treated with post-operative corticosteroids.	2	II-, good quality	Strong
In high-risk patients, e.g., pre-existing glaucoma, the intraocular pressure should be monitored in the early post-operative period and appropriate pressure-lowering agents given.	2	III, good quality	Strong
There is no evidence that visual outcome is improved by routine use of prophylactic non-steroidal anti-inflammatory drugs at ≥3 months after cataract surgery.	2	II+, moderate quality	Strong
Offer eye protection for patients whose eye shows residual effects of anaesthesia at time of discharge after surgery.	1	No evidence was identified	Strong
Consider collecting visual function and quality of life data for entry into an electronic dataset.	1	Low	Discretionary
Do not offer face-to-face first-day review to patients who had uncomplicated cataract surgery.	1	Low	Strong
If endophthalmitis is suspected, refer to a retina specialist to review the patient within 24 h. If review within 24 h is not possible, perform an intravitreal tap and inject of antibiotics.	2	I-, good quality	Strong
When unacceptable refractive error results after lens implantation, the risks of further surgery must be weighed against use of spectacles or contact lens correction.	2	III, good quality	Strong
Patients with uveitis generally require greater frequency and duration of topical anti-inflammatory treatment and should be monitored closely.	2	III, good quality	Strong
YAG laser capsulotomy is indicated for posterior capsular opacification where there is a functional impact. The eye should be inflammation-free and the IOL stable prior to performing a YAG capsulotomy.	2	III, good quality	Strong
High frequency post-operative antibiotics are recommended.	3	III	-
Topical steroids or non-steroidal anti-inflammatory drugs to reduce post-operative inflammation is recommended.	3	IV	-
Ophthalmologists should be aware of resistance to several antibiotics such as penicillin and fluoroquinolones for treatment of postoperative staphylococcal endophthalmitis.	3	III	-
Ophthalmologists should be aware of toxic anterior segment syndrome and its predisposing factors.	3	III	-
To evaluate patient satisfaction, standard questionnaires such as the VF14 will provide greater insight into patient satisfaction than visual acuity assessment.	3	IV	-

*** Clinical Practice Guidelines:** CPG 1: National Institute for Health and Care Excellence (NICE), 2017, Cataracts in adults; management [14]. CPG 2: American Academy of Ophthalmology, 2016, Cataract in the Adult Eye Preferred Practice Pattern [15]. CPG 3: Rajavi et al., 2015, Customized clinical practice guidelines for management of adult cataract in Iran [16]. CPG 4: The Royal College of Ophthalmologists and Clinical Council for Eye Health Commissioning, 2018, Commissioning guide: Adult cataract surgery [17]. **** Definitions of *quality* for each CPG:** CPG 1: “GRADE was used to assess the quality of evidence for the selected outcomes as specified in ‘The guidelines manual (2014)’ [32]. Where RCTs are available, these are initially rated as high quality and the quality of the evidence for each outcome was downgraded or not from this initial point. If non-RCT evidence was included for intervention-type systematic reviews, then these are initially rated as low-quality and the quality of the evidence for each outcome was downgraded or not from this point”. CPG 2: “To rate individual studies, a scale based on SIGN [33] is used. The definitions and levels of evidence to rate individual studies are as follows: I++ High-quality meta-analyses, systematic reviews of randomized controlled trials (RCTs), or RCTs with a very low risk of bias. I+ Well-conducted meta-analyses, systematic reviews of RCTs, or RCTs with a low risk of bias. I- Meta-analyses, systematic reviews of RCTs, or RCTs with a high risk of bias. II++ High-quality systematic reviews of case–control or cohort studies High-quality case–control or cohort studies with a very low risk of confounding or bias and a high probability that the relationship is causal. II+ Well-conducted case–control or cohort studies with a low risk of confounding or bias and a moderate probability that the relationship is causal. II- Case-control or cohort studies with a high risk of confounding or bias and a significant risk that the relationship is not causal. III Nonanalytic studies (e.g., case reports, case series). The body of evidence quality ratings are defined by GRADE [18] as follows: Good quality: Further research is very unlikely to change our confidence in the estimate of effect. Moderate quality: Further research is likely to have an important impact on our confidence in the estimate of effect and may change the estimate. Insufficient quality: Further research is very likely to have an important impact on our confidence in the estimate of effect and is likely to change the estimate. Any estimate of effect is very uncertain”. CPG 3: I Randomized clinical trials; Systematic reviews; Meta-analysis. II Controlled clinical study without randomization at least one; Well-designed cohort study; Well-designed case–control; Cross sectional study. III Surveys; descriptive; case series studies. IV Experts opinion; consensus. ***** Definitions of *Strong* and *Discretionary*:** CPG 1: Strong recommendation: “We use ‘offer’ (and similar words such as ‘refer’ or ‘advise’) when we are confident that, for the vast majority of patients, an intervention will do more good than harm, and be cost effective. We use similar forms of words (for example, ‘Do not offer…’) when we are confident that an intervention will not be of benefit for most patients’. Discretionary recommendation: ‘We use ‘consider’ when we are confident that an intervention will do more good than harm for most patients, and be cost effective, but other options may be similarly cost effective. The choice of intervention, and whether or not to have the intervention at all, is more likely to depend on the patient’s values and preferences than for a strong recommendation, and so the healthcare professional should spend more time considering and discussing the options with the patient”. CPG 2: Strong recommendation: “Used when the desirable effects of an intervention clearly outweigh the undesirable effects or clearly do not”. Discretionary recommendation: “Used when the trade-offs are less certain—either because of low-quality evidence or because evidence suggests that desirable and undesirable effects are closely balanced”.
