# Supplementary material for: A Systematic Review of Clinical Practice Guidelines for Cataract: Evidence to Support the Development of the WHO Package of Eye Care Interventions
Source: Vision (Basel). 2022 Jun 20;6(2):36. doi: 10.3390/vision6020036 (PMC9227019; doi:10.3390/vision6020036)
Supplement: Supplementary file 1 [file vision-06-00036-s001.zip › vision-1710954-supplementary.pdf]

Supplementary Materials

Supplement to: Zhang JH, Ramke J, Lee CN, et al. A systematic review of Clinical Practice Guidelines for cataract: Evidence to support the development of the WHO Package of Eye Care Interventions.

Supplementary Material S1: PRISMA reporting guidelines

Section and Topic	Item #	Checklist item	Location where item is reported
**TITLE**			
Title	1	Identify the report as a systematic review.	Title; abstract
**ABSTRACT**			
Abstract	2	See the PRISMA 2020 for Abstracts checklist.	Abstract
**INTRODUCTION**			
Rationale	3	Describe the rationale for the review in the context of existing knowledge.	Introduction
Objectives	4	Provide an explicit statement of the objective(s) or question(s) the review addresses.	Introduction
**METHODS**			
Eligibility criteria	5	Specify the inclusion and exclusion criteria for the review and how studies were grouped for the syntheses.	Methods (Eligibility Criteria); Table 1
Information sources	6	Specify all databases, registers, websites, organisations, reference lists and other sources searched or consulted to identify studies. Specify the date when each source was last searched or consulted.	Supplementary Material S3; Methods (Information Sources and Search)
Search strategy	7	Present the full search strategies for all databases, registers and websites, including any filters and limits used.	Supplementary Material S2
Selection process	8	Specify the methods used to decide whether a study met the inclusion criteria of the review, including how many reviewers screened each record and each report retrieved, whether they worked independently, and if applicable, details of automation tools used in the process.	Methods (Selection of Sources of Evidence and Appraisal)

Section and Topic	Item #	Checklist item	Location where item is reported
Data collection process	9	Specify the methods used to collect data from reports, including how many reviewers collected data from each report, whether they worked independently, any processes for obtaining or confirming data from study investigators, and if applicable, details of automation tools used in the process.	Methods (Selection of Sources of Evidence and Appraisal)
Data items	10a	List and define all outcomes for which data were sought. Specify whether all results that were compatible with each outcome domain in each study were sought (e.g. for all measures, time points, analyses), and if not, the methods used to decide which results to collect.	Methods (Data Charting and Data Items)
	10b	List and define all other variables for which data were sought (e.g. participant and intervention characteristics, funding sources). Describe any assumptions made about any missing or unclear information.	Methods (Data Charting and Data Items)
Study risk of bias assessment	11	Specify the methods used to assess risk of bias in the included studies, including details of the tool(s) used, how many reviewers assessed each study and whether they worked independently, and if applicable, details of automation tools used in the process.	N/A
Effect measures	12	Specify for each outcome the effect measure(s) (e.g. risk ratio, mean difference) used in the synthesis or presentation of results.	N/A
Synthesis methods	13a	Describe the processes used to decide which studies were eligible for each synthesis (e.g. tabulating the study intervention characteristics and comparing against the planned groups for each synthesis (item #5)).	Methods (Eligibility Criteria)
	13b	Describe any methods required to prepare the data for presentation or synthesis, such as handling of missing summary statistics, or data conversions.	Methods (Synthesis of Results)
	13c	Describe any methods used to tabulate or visually display results of individual studies and syntheses.	Methods (Synthesis of Results)
	13d	Describe any methods used to synthesize results and provide a rationale for the choice(s). If meta-analysis was performed, describe the model(s), method(s) to identify the presence and extent of statistical heterogeneity, and software package(s) used.	Methods (Synthesis of Results)
	13e	Describe any methods used to explore possible causes of heterogeneity among study results (e.g. subgroup analysis, meta-regression).	N/A
	13f	Describe any sensitivity analyses conducted to assess robustness of the synthesized results.	N/A

Section and Topic	Item #	Checklist item	Location where item is reported
Reporting bias assessment	14	Describe any methods used to assess risk of bias due to missing results in a synthesis (arising from reporting biases).	N/A
Certainty assessment	15	Describe any methods used to assess certainty (or confidence) in the body of evidence for an outcome.	Strength of recommendation: Methods (Synthesis of Results)
**RESULTS**			
Study selection	16a	Describe the results of the search and selection process, from the number of records identified in the search to the number of studies included in the review, ideally using a flow diagram.	Figure 1
	16b	Cite studies that might appear to meet the inclusion criteria, but which were excluded, and explain why they were excluded.	Figure 1
Study characteristics	17	Cite each included study and present its characteristics.	Results; Appendix A
Risk of bias in studies	18	Present assessments of risk of bias for each included study.	N/A
Results of individual studies	19	For all outcomes, present, for each study: (a) summary statistics for each group (where appropriate) and (b) an effect estimate and its precision (e.g. confidence/credible interval), ideally using structured tables or plots.	N/A
Results of syntheses	20a	For each synthesis, briefly summarise the characteristics and risk of bias among contributing studies.	Results; Appendix A
	20b	Present results of all statistical syntheses conducted. If meta-analysis was done, present for each the summary estimate and its precision (e.g. confidence/credible interval) and measures of statistical heterogeneity. If comparing groups, describe the direction of the effect.	N/A
	20c	Present results of all investigations of possible causes of heterogeneity among study results.	N/A
	20d	Present results of all sensitivity analyses conducted to assess the robustness of the synthesized results.	N/A

Section and Topic	Item #	Checklist item	Location where item is reported
Reporting biases	21	Present assessments of risk of bias due to missing results (arising from reporting biases) for each synthesis assessed.	N/A
Certainty of evidence	22	Present assessments of certainty (or confidence) in the body of evidence for each outcome assessed.	Strength of recommendation: Methods (Synthesis of Results)
**DISCUSSION**			
Discussion	23a	Provide a general interpretation of the results in the context of other evidence.	Discussion
	23b	Discuss any limitations of the evidence included in the review.	Discussion
	23c	Discuss any limitations of the review processes used.	Discussion
	23d	Discuss implications of the results for practice, policy, and future research.	Discussion
**OTHER INFORMATION**			
Registration and protocol	24a	Provide registration information for the review, including register name and registration number, or state that the review was not registered	Protocol published but not registered
	24b	Indicate where the review protocol can be accessed, or state that a protocol was not prepared.	Methods
	24c	Describe and explain any amendments to information provided at registration or in the protocol.	N/A
Support	25	Describe sources of financial or non-financial support for the review, and the role of the funders or sponsors in the review.	Funding statement
Competing interests	26	Declare any competing interests of review authors.	Competing interests
Availability of data, code and other materials	27	Report which of the following are publicly available and where they can be found: template data collection forms; data extracted from included studies; data used for all analyses; analytic code; any other materials used in the review.	Data sharing statement

Supplementary Material S2: Search strategy for academic databases

MEDLINE
1 exp eye diseases/ (557476)
2 visually impaired persons/ (2391)
3 or/1-2 (558065)
4 Practice Guidelines as Topic/ (115227)
5 Guidelines as Topic/ (39163)
6 Practice Guideline/ (26462)
7 ((clinical or practice) adj2 guideline$).tw. (41838)
8 or/4-7 (202728)
9 3 and 8 (2180)
10 exp case reports/ (2078744)
11 case report$.tw. (356889)
12 or/10-11 (2160660)
13 9 not 12 (2112)
14 limit 13 to english language (1676)
15 limit 14 to yr="2009 -Current" (949)
16 limit 15 to (address or autobiography or bibliography or biography or classical article or clinical trial, veterinary or clinical trials, veterinary as topic or comment or directory or editorial or "expression of concern" or festschrift or historical article or interactive tutorial or interview or letter or news or newspaper article or observational study, veterinary or personal narrative or portrait or video-audio media or webcast) (113)
17 15 not 16 (836)

Embase
1 exp eye disease/cn, di, dm, ep, et, pc, rh, su, th [Congenital Disorder, Diagnosis, Disease Management, Epidemiology, Etiology, Prevention, Rehabilitation, Surgery, Therapy] (314642)
2 visual impairment/cn, di, dm, ep, et, pc, rh, su, th [Congenital Disorder, Diagnosis, Disease Management, Epidemiology, Etiology, Prevention, Rehabilitation, Surgery, Therapy] (6819)
3 or/1-2 (314642)
4 practice guidelines/ (405820)
5 ((clinical or practice) adj2 guideline$).tw. (60447)
6 or/4-5 (428306)
7 3 and 6 (3082)
8 exp case report/ (2347870)
9 case report$.tw. (464572)
10 or/8-9 (2402846)
11 7 not 10 (2932)
12 limit 11 to english language (2574)
13 limit 12 to yr="2009 -Current" (1346)
14 limit 13 to (conference abstract or conference paper or editorial or letter or note or short survey or tombstone) (257)
15 13 not 14 (1089)

Global Health
1 exp eye diseases/ (32535)
2 people with visual impairment/ (170)
3 or/1-2 (32658)
4 exp guideline/ (47207)
5 ((clinical or practice) adj2 guideline$).tw. (4484)

6 or/4-5 (49229)
7 3 and 6 (333)
8 case reports/ (86862)
9 case report$.tw. (94202)
10 8 or 9 (94202)
11 7 not 10 (329)
12 limit 11 to english language (279)
13 limit 12 to yr="2009 -Current" (197)

<u>CINAHL</u>
S5 S1 AND S4 Limiters - Published Date: 20090101-20200231; English Language; Exclude MEDLINE records
S4 S2 OR S3
S3 (TX clinical OR TX practice) N2 (guideline*)
S2 MM "Practice Guidelines"
S1 MH "Eye Diseases+"

<u>WHO Global Index Medicus</u>
SEARCH 1
(ab:(practice guideline)) AND (ab:(eye OR vision OR visual OR blindness))
SEARCH 2
(mh:(eye diseases)) AND (mh:(practice guideline))

Supplementary Material S3: Guideline databases and the websites of professional ophthalmology and optometry associations searched.

Guideline databases

1. Guidelines International Network
2. National Institute for Clinical Excellence (UK)
3. eGuidelines
4. Guideline Central
5. ECRI Guidelines Trust Database
6. Australian National Health and Medical Research Council clinical practice guidelines
7. Canadian Medical Association Infobase of Clinical Practice Guidelines
8. Trip Database
9. WHO guidelines
10. US Preventive Services Task Force Guideline
11. Scottish Intercollegiate Guidelines Network (SIGN)

Professional association websites

1. College of Optometrists (UK)
2. Pan American Association of Ophthalmology
3. Eastern Mediterranean Council of Optometry
4. European Society of Ophthalmology
5. World Council of Optometry
6. American Academy of Ophthalmology
7. American Optometric Association
8. Asia Pacific Council of Optometry
9. International Council of Ophthalmology
10. Royal Australian and New Zealand College of Ophthalmologists
11. Royal College of Ophthalmologists
12. European Society of Cataract and Refractive Surgeons
13. American Society of Cataract and Refractive Surgeons
14. European Vitreoretinal Society
15. African Ophthalmology Council
16. Asia Pacific Academy of Ophthalmology
